# Supplementary material for: How Many Hungarian Consumers Choose Lactose- and Gluten-Free Food Products Even When They Do Not Necessarily Need to?
Source: Foods. 2023 Oct 31;12(21):3984. doi: 10.3390/foods12213984 (PMC10649371; doi:10.3390/foods12213984)
Supplement: Supplementary file 1 [file foods-12-03984-s001.zip › foods-2638786-supplementary.pdf]

SORSZÁM □ □ □ □ **Állatorvostudományi Egyetem** Időpont:

Felmérés az egészséges táplálkozást támogató élelmiszerekről 2018

Tisztelt Válaszadó! Rövid kérdőívünk segítségével szeretnénk megismerni az egészséges táplálkozást támogató „egészséges élelmiszerekkel" kapcsolatos véleményét, ismereteit, fogyasztói szokásait. Kérdőívünk név nélküli, az Ön személye semmilyen módon nem azonosítható be. **Segítségét köszönjük! dr. Szabó Erika, Szakos Dávid (szakos.david@univet.hu)**

Ön milyen mértékben ért egyet az alábbi állításokkal?
Kérjük, értékeljen 1-től 5-ig. 1: egyáltalán nem értek egyet; 5: teljes mértékben egyetértek

Folyamatosan tájékozódom, tanulok az élelmiszerekről, a táplálkozásról	①②③④⑤
Tudatosan választok márkát, gyártót, ha élelmiszerről van szó	①②③④⑤
Alaposan elolvasom a termékek címkéjét	①②③④⑤
A táplálkozás közvetlen hatást gyakorol az egészségi állapotra	①②③④⑤
Fontos számomra, hogy egészségesen táplálkozzak	①②③④⑤
Az egészségesebb élelmiszerek kevésbé finomak	①②③④⑤
Idősebb korban fokozottan számít a táplálkozás az egészség szempontjából	①②③④⑤
Egészséges táplálkozással sokat tehetünk az időskori betegségek megelőzéséért	①②③④⑤
Egy „egészséges élelmiszerért" hajlandó vagyok többet fizetni	①②③④⑤
Az „egészségesebb élelmiszerek" drágábbak	①②③④⑤
Érdekel az egészséges táplálkozás	①②③④⑤
Érdekelnek az életmóddal kapcsolatos tudományos kérdések	①②③④⑤
Többnyire otthon elkészített ételeket eszem	①②③④⑤
Gyakran eszem étteremben	①②③④⑤
Gyakran fogyasztok laktózmentes (tejcukormentes) élelmiszereket	①②③④⑤
Gyakran fogyasztok gluténmentes élelmiszereket	①②③④⑤
Gyakran fogyasztok cukormentes élelmiszereket	①②③④⑤
Gyakran fogyasztok szénhidrátmentes vagy szénhidrát-szegény élelmiszereket	①②③④⑤
Véleményem szerint a tejtermékek segítik a fogyást	①②③④⑤
Az élelmiszerek íze a legfontosabb szempont, amikor vásárolok	①②③④⑤
Az élelmiszerek ára a legfontosabb szempont, amikor vásárolok	①②③④⑤
Az élelmiszerek márkája a legfontosabb szempont, amikor vásárolok	①②③④⑤
Az élelmiszerek csomagolása a legfontosabb szempont, amikor vásárolok	①②③④⑤
Az élelmiszerek hazai eredete a legfontosabb szempont, amikor vásárolok	①②③④⑤
Az élelmiszerek minősége a legfontosabb szempont, amikor vásárolok	①②③④⑤
Az élelmiszerek tápanyag-összetétele a legfontosabb szempont, amikor vásárolok	①②③④⑤
Az egészséges étrendbe való jó beilleszthetőség a fő vásárlási szempontom	①②③④⑤
Véleményem szerint sok tejtermékek fogyasztásával könnyen elhízhatunk	①②③④⑤

Ön szerint mit jelent az „egészséges élelmiszer" kifejezés? Kérjük, legalább egy-két olyan szót említsen, amely a fogalomról eszébe jut.

Amikor élelmiszert vásárol, mennyire jelent előnyt, ha az alábbi feliratokkal találkozik?
Kérjük, értékeljen 1-től 5-ig. 1: egyáltalán nem fontos számomra; 5: nagyon fontos számomra

Energiaszegény	①②③④⑤
Csökkentett energiatartalmú	①②③④⑤
Energiamentes	①②③④⑤
Zsírszegény	①②③④⑤
Zsírmentes	①②③④⑤
Telített zsírban szegény	①②③④⑤
Telített zsírtól mentes	①②③④⑤
Csökkentett telítettzsírsav-tartalmú	①②③④⑤
Hozzáadott cukrot nem tartalmaz	①②③④⑤
Alacsony cukortartalmú	①②③④⑤
Cukormentes	①②③④⑤
Nátriumszegény	①②③④⑤
Sószegény	①②③④⑤
Kifejezetten nátriumszegény	①②③④⑤
Kifejezetten sószegény	①②③④⑤
Nátriummentes	①②③④⑤
Sómentes	①②③④⑤
Élelmirost-forrás	①②③④⑤
Élelmi rostban gazdag	①②③④⑤
Fehérjeforrás	①②③④⑤
Fehérjében gazdag	①②③④⑤
Megnövelt fehérjetartalom	①②③④⑤
Vitamin-forrás	①②③④⑤
Vitaminban gazdag	①②③④⑤
Kalcium-forrás	①②③④⑤
Természetes kalcium-forrás	①②③④⑤
Kalciumban gazdag	①②③④⑤
Kalciumban természetesen gazdag	①②③④⑤
Pektint tartalmaz	①②③④⑤
Alacsony szénhidráttartalmú	①②③④⑤
Laktózmentes	①②③④⑤
Gluténmentes	①②③④⑤
Élőflórás	①②③④⑤
Teljes kiőrlésű	①②③④⑤
Gyógynövény tartalmú	①②③④⑤
Antioxidáns tartalmú	①②③④⑤
Prebiotikus	①②③④⑤
Probiotikus	①②③④⑤
Light/lite	①②③④⑤

Ön szerint milyen típusú élelmiszerek fogyasztásával tesz a legtöbbet az egészségéért?
Kérjük, értékeljen 1-től 5-ig. 1: ezzel az élelmiszerrel egyáltalán nem; 5: ezzel az élelmiszerrel kifejezetten igen

Nyers gyümölcs	①②③④⑤
Joghurt	①②③④⑤
Tej	①②③④⑤
Kefir	①②③④⑤
Sajt	①②③④⑤
Vaj	①②③④⑤
Nyers zöldség	①②③④⑤
Tejföl	①②③④⑤
Túró	①②③④⑤
Gyógynövény tartalmú élelmiszerek	①②③④⑤
Méz	①②③④⑤
Olajos magvak, müzlik	①②③④⑤
Margarin	①②③④⑤
Zöldség alapú élelmiszerek	①②③④⑤
Gyümölcs alapú élelmiszerek	①②③④⑤
Gomba alapú élelmiszerek	①②③④⑤
Lekvár	①②③④⑤
Pékáruk	①②③④⑤
Hústermékek (pl. kolbász)	①②③④⑤
Baromfihúsból készült ételek	①②③④⑤
Sertéshúsból készült ételek	①②③④⑤
Tojás és tojás alapú élelmiszerek	①②③④⑤
Haltermékek, halételek	①②③④⑤
Gyümölcslevek, zöldséglevek	①②③④⑤
Teák	①②③④⑤

Az Ön számára mennyire fontos, hogy egy „egészséges élelmiszer" rendelkezzen az alábbi tulajdonságokkal?
Kérjük, értékeljen 1-től 5-ig. 1: egyáltalán nem fontos ez a tulajdonság; 5: nagyon fontos tulajdonság

Kistermelő által előállított	①②③④⑤
Biotermék	①②③④⑤
Hagyományos jellegű termék	①②③④⑤
Hazai termék	①②③④⑤
Védjeggyel ellátott	①②③④⑤
Ismert gyártó által előállított	①②③④⑤
Modern technológiával készült	①②③④⑤
Genetikai módosítástól mentes	①②③④⑤
Állatjóléti szempontok figyelembevételével készült	①②③④⑤
Hazai kutatók munkájának eredményeképpen jött létre	①②③④⑤
A hazai élelmiszerlánc-biztonsági hatóság (NÉBIH) vizsgálatai szerint	①②③④⑤

Az Ön háztartása hol szokott bevásárolni?

Osztályozzon 1-től 5-ig: 5: Nagyon jellemző – 1: Egyáltalán nem jellemző

Nagyobb boltban (pl. szupermarket, hipermarket)	①②③④⑤
Kisboltban	①②③④⑤
Közvetlenül a termelőnél (például piacon)	①②③④⑤
Interneten	①②③④⑤
Saját magam termesztem, vagy ajándékba kapom	①②③④⑤
Egyéb, éspedig:	

Melyik állítás igaz és melyik hamis? Jelölje be a megfelelő választ.

Állítás	Igaz	Hamis
A tartós tej jellegzetes íze a benne lévő tartósítószernek köszönhető	❑	❑
A boltban kapható tejet hőkezelik	❑	❑
A hagyományos élelmiszerek génmentesek	❑	❑
Ha egy termékben baktériumok vannak, akkor az már nem biztonságos	❑	❑
A természetes/bio élelmiszerek kockázatmentesek	❑	❑
A konzervekben van tartósítószer	❑	❑

Ön szerint mik a laktózérzékenység tünetei?

Ön szerint milyen módon tud tejtermékeket fogyasztani panaszmentesen egy laktózérzékeny ember?

Az alábbi életmóddal kapcsolatos állítások közül melyikek igazak Önre? *Több választ is megjelölhet*

❑ Rendszeresen sportolok
❑ Szeretnék leadni néhány kilót, és ezért teszek is
❑ Szeretném, hogy jobb legyen az állóképességem, erőnlétem, és ezért teszek is
❑ Túl keveset alszom
❑ Stresszes az életmódom
❑ Alapvetően egészségesnek érzem magam
❑ Kevés időt tudok az étkezésre, ételkészítésre fordítani
❑ Úgy érzem, hogy túl keveset mozgok
❑ Az átlagosnál jobban odafigyelek az étrendemre
❑ Napi rendszerességgel dohányzom
❑ Igyekszem kevesebb alkoholt fogyasztani
❑ Fogyasztok étrend-kiegészítőket

Az Ön számára mely problémák aggasztóak, vagy melyekben érintett? Elkerülésükért anyagi kiadásokat is vállalna?

**Probléma megnevezése**	**Ez a probléma nem aggaszt/ nem érint**	**Aggaszt/érint, de egyelőre nem vállalok anyagi kiadásokat az elkerülése érdekében**	**Aggaszt/érint, elkerülése érdekében anyagi kiadásokat is vállalnék/vállalok**
Szív és érrendszeri betegségek	❑	❑	❑
Magas koleszterinszint	❑	❑	❑
Daganatos betegség	❑	❑	❑
Hangulatzavar, alvászavar	❑	❑	❑
Légúti betegség	❑	❑	❑
Bőrbetegség, ekcéma	❑	❑	❑
Memóriazavarok, dekoncentráció	❑	❑	❑
Emésztési problémák	❑	❑	❑
Migrén	❑	❑	❑
Cukorbetegség	❑	❑	❑
Ízületi betegségek	❑	❑	❑
Csontritkulás	❑	❑	❑
Legyengült immunrendszer	❑	❑	❑
Allergia	❑	❑	❑
Fogászati problémák	❑	❑	❑
Hormonális problémák	❑	❑	❑
Látásproblémák	❑	❑	❑
Laktózérzékenység	❑	❑	❑
Gluténérzékenység	❑	❑	❑

Ha az Ön által megjelölt problémák többféle módon is kezelhetők/megelőzhetők lennének, az alábbi lehetőségek közül melyiket választaná legszívesebben? *Több választ is megjelölhet*

Kezelési/ Megelőzési mód / Probléma megnevezése	Gyógyszer	Étrend-kiegészítő	Egészségesebb élelmiszerek	Életmód-változtatás
Szív és érrendszeri betegségek	❑	❑	❑	❑
Magas koleszterinszint	❑	❑	❑	❑
Daganatos betegség	❑	❑	❑	❑
Hangulatzavar, alvászavar	❑	❑	❑	❑
Légúti betegség	❑	❑	❑	❑
Bőrbetegség, ekcéma	❑	❑	❑	❑
Memóriazavarok, dekoncentráció	❑	❑	❑	❑
Emésztési problémák	❑	❑	❑	❑
Migrén	❑	❑	❑	❑
Cukorbetegség	❑	❑	❑	❑
Ízületi betegségek	❑	❑	❑	❑
Csontritkulás	❑	❑	❑	❑
Legyengült immunrendszer	❑	❑	❑	❑
Allergia	❑	❑	❑	❑
Fogászati problémák	❑	❑	❑	❑
Hormonális problémák	❑	❑	❑	❑
Látásproblémák	❑	❑	❑	❑
Laktózérzékenység	❑	❑	❑	❑
Gluténérzékenység	❑	❑	❑	❑

A táplálkozással és élelmiszerekkel kapcsolatos információk honnan jutnak el Önhöz?
Osztályozzon 1-től 5-ig: 5: Innen kifejezetten igen – 1: Innen egyáltalán nem

Televízió	①②③④⑤
Internetes híroldalak és blogok	①②③④⑤
Internetes közösségi oldalak	①②③④⑤
Rádió	①②③④⑤
Család, ismerősök, rokonok	①②③④⑤
Nyomtatott újságok, magazinok	①②③④⑤
Iskola, egyetem, továbbképzés	①②③④⑤
Egyéb, éspedig:	

Van-e az Ön családjában laktózérzékeny? ❑ Igen, én ❑ Igen, családtag ❑ Nincs

Van-e az Ön családjában gluténérzékeny? ❑ Igen, én ❑ Igen, családtag ❑ Nincs

Amennyiben Ön fogyaszt laktózmentes termékeket, járt-e emiatt orvosnál? ❑ Igen ❑ Nem

Amennyiben Ön fogyaszt gluténmentes, járt-e emiatt orvosnál? ❑ Igen ❑ Nem

DEMOGRÁFIA

A kutatási eredmények feldolgozása érdekében kérjük, válaszoljon az alábbi, az Ön személyével kapcsolatos kérdéseinkre – a kutatás **név nélküli** és a feldolgozás módja garantálja, hogy **az Ön személye nem beazonosítható** a felmérésben.

Neme: ❑ Nő ❑ Férfi

Születési évszám: __ __ __ __

Lakóhelye (település típusa): ❑Község/falu ❑Város ❑Főváros

Lakóhelye (megye): ________________

Az Ön befejezett legmagasabb iskolai végzettsége
❑ Általános iskola (8 vagy kevesebb osztály)
❑ Szakiskola (szakmunkás végzettség)
❑ Érettségi
❑ Főiskolai, egyetemi oklevél

Az Ön gazdasági státusza (kérjük, csak egyet jelöljön meg)
❑ Aktív dolgozó (munkavállaló)
❑ Vállalkozó (önfoglalkoztató)
❑ Nyugdíjas, rokkant nyugdíjas
❑ Munkakereső
❑ Háztartásbeli, GYES-GYED
❑ Diák

A háztartásában lakók száma: Én **+_____fő**

Van-e az Ön háztartásában 15 éven aluli gyermek? ❑ Van ❑ Nincs

Van-e olyan ok, amely miatt Önnek különleges étrendre van szüksége? *Több választ is megjelölhet!*
❑ Igen, cukorbetegség (diabetes)
❑ Igen, lisztérzékenység (cöliákia)
❑ Igen, tejfehérje érzékenység/allergia
❑ Igen, fogyni szeretne
❑ Igen, magas vérnyomás
❑ Igen, sportosabb, izmosabb szeretne lenni
❑ Igen, vegetáriánus/vegán
❑ Igen, valamilyen speciális diétát követ (pl. lúgosító, paleolit, nyers étel)
❑ Igen, egyéb okból kifolyólag:_________________
❑ Nincs ilyen ok, de a családomban van
❑ Nincs ilyen ok

Ki az Ön háztartásában az „élelmiszerfelelős” vagyis ki hozza meg az élelmiszervásárlással, ételkészítéssel kapcsolatos döntéseket?
❑ Ön ❑ Közösen vagy munkamegosztásban ❑ Más személy

Az Ön háztartásának jövedelemszintjére melyik állítás igaz?
❑ Még a legszükségesebb dolgok megvásárlása is nehézségekbe ütközik
❑ Szerényen, de megélünk
❑ Jövedelemszintünk átlagos, amit fontosnak tartunk, meg tudjuk venni
❑ Jövedelemszintünk átlag feletti
❑ Jövedelemszintünk kiemelkedő

Testtömege: …………………… kg **Testmagassága: …………………… cm**
